# Comparison of human adult stem cells from adipose tissue and bone marrow in the treatment of experimental autoimmune encephalomyelitis

**DOI:** 10.1186/scrt391

**Published:** 2014-01-09

**Authors:** Julie A Semon, Catherine Maness, Xiujuan Zhang, Steven A Sharkey, Marc M Beuttler, Forum S Shah, Amitabh C Pandey, Jeffrey M Gimble, Shijia Zhang, Brittni A Scruggs, Amy L Strong, Thomas A Strong, Bruce A Bunnell

**Affiliations:** 1 Center for Stem Cell Research and Regenerative Medicine, School of Medicine, Tulane University, 1430 Tulane Avenue, SL-99, New Orleans, LA 70112, USA; 2 Department of Cell and Molecular Biology, School of Science and Engineering, Tulane University, 6400 Freret Street, New Orleans, LA 70118, USA; 3 Department of Pharmacology, School of Medicine, Tulane University, 1430 Tulane Avenue, SL-83, New Orleans, LA 70112, USA; 4 Stem Cell Biology Laboratory, Pennington Biomedical Research Center, Louisiana State University System, 6400 Perkins Road, Baton Rouge, LA 70808, USA

## Abstract

**Introduction:**

While administration of *ex vivo* culture-expanded stem cells has been used to study immunosuppressive mechanisms in multiple models of autoimmune diseases, less is known about the uncultured, nonexpanded stromal vascular fraction (SVF)-based therapy. The SVF is composed of a heterogeneous population of cells and has been used clinically to treat acute and chronic diseases, alleviating symptoms in a range of tissues and organs.

**Methods:**

In this study, the ability of human SVF cells was compared with culture-expanded adipose stem cells (ASCs) and bone-derived marrow stromal cells (BMSCs) as a treatment of myelin oligodendrocyte glycoprotein (35–55)-induced experimental autoimmune encephalitis in C57Bl/6J mice, a well-studied multiple sclerosis model (MS). A total of 1 × 10^6^ BMSCs, ASCs, or SVF cells were administered intraperitoneally concomitantly with the induction of disease. Mice were monitored daily for clinical signs of disease by three independent, blinded investigators and rated on a scale of 0 to 5. Spinal cords were obtained after euthanasia at day 30 and processed for histological staining using luxol fast blue, toluidine blue, and hematoxylin and eosin to measure myelin and infiltrating immune cells. Blood was collected from mice at day 30 and analyzed by enzyme-linked immunosorbent assay to measure serum levels of inflammatory cytokines.

**Results:**

The data indicate that intraperitoneal administration of all cell types significantly ameliorates the severity of disease. Furthermore, the data also demonstrate, for the first time, that the SVF was as effective as the more commonly cultured BMSCs and ASCs in an MS model. All cell therapies also demonstrated a similar reduction in tissue damage, inflammatory infiltrates, and sera levels of IFNγ and IL-12. While IFNγ levels were reduced to comparable levels between treatment groups, levels of IL-12 were significantly lower in SVF-treated than BMSC-treated or ASC-treated mice.

**Conclusions:**

Based on these data, it is evident that SVF cells have relevant therapeutic potential in an animal model of chronic MS and might represent a valuable tool for stem cell-based therapy in chronic inflammatory disease of the central nervous system. SVF offers advantages of direct and rapid isolation procedure in a xenobiotic-free environment.

## Introduction

Adult marrow stromal cells, also referred to as mesenchymal stromal/stem cells (MSCs), have been used for cell therapy and in tissue engineering because of their ability to differentiate into multiple mesenchymal and nonmesenchymal lineages *in vitro*, their immune modulatory effects both *in vivo* and *in vitro*, and their ability to home to sites of tissue damage [1,2]. MSCs have been isolated from several tissues, including the bone marrow, adipose tissue, umbilical cord blood, liver, synovium, skeletal muscle, kidney, skin tissue, lung, and intestinal tract [3-7]. Currently, MSCs derived from different sources are being utilized in clinical studies for the treatment of several diseases and injuries such as myocardial infarction, osteogenesis imperfecta, graft-versus-host disease, Crohn’s disease, spinal cord injury, multiple sclerosis (MS), and diabetes [8,9]. Although the optimal dosage of MSCs in clinical applications is still undetermined, the general criteria is 1 × 10^6^ to 2 × 10^6^ MSCs/kg body weight per injection, which makes using primary MSCs difficult for clinical use [10].

Since a large number of cells are often required for clinical and basic research studies, the primary cultures need to be *ex vivo* expanded for multiple passages on tissue culture substrates. Typically, MSCs can undergo 24 to 40 population doublings in culture before reaching senescence [11,12]. However, after the initial culture period, MSCs progressively lose their multipotentiality [13,14]. Fetal bovine serum (FBS), which contains a high content of growth factors as well as nutritional and physiochemical compounds required for cell maintenance and growth, is typically used at 10 to 20% (v/v) in media. Despite its common use, FBS is ill-defined and presents numerous potential problems for the expansion of MSCs [15-19]. Owing to the concerns of using FBS, particularly for clinical therapy, attempts have been made to develop defined serum-free media. Most of these media have been inadequate, with cells growing at a slower proliferative rate, with minimal passages, and still using serum-based media for initial isolation and expansion phases [20,21].

The frequency of MSCs in bone marrow is very low. MSCs represent 0.01 to 0.001% of human bone marrow mononuclear cells [22,23]. However, recent studies report that MSCs are found at a higher frequency in adipose tissue, yielding 100 to 500 times more cells per tissue volume [24,25]. These adipose stem cells (ASCs) have similar self-renewal abilities, common surface epitopes, growth kinetics, and cytokine expression profiles to bone-derived marrow stromal cells (BMSCs), but they are not associated with the morbidity, pain, or low yield [3,5,26]. In addition, recent data indicate that ASCs are potently immunomodulatory, induce angiogenesis, and are multipotent, making them an appealing alternative to BMSCs [24-29].

Despite the promise of ASCs, the need for *ex vivo* cellular expansion is still a significant obstacle. A more direct procedure, for which adipose tissue is uniquely suited, is the administration of a nonexpanded cellular fraction, the stromal vascular fraction (SVF). Adipose tissue is easy to obtain in large quantities and should, therefore, be able to provide a readily available source of stromal stem cells in numbers sufficient to use clinically or to study their biology without culturing cells. Anti-inflammatory and regenerative effects of nonexpanded SVF cells have yielded promising results in canine osteoarthritis and equine tendon ligament injuries [30]. With encouraging outcomes in clinical trials [30], it is essential to examine the effects and mechanisms of SVF, especially in comparison with BMSC and ASC therapy, in models of autoimmune disease.

MS is an autoimmune disease characterized by inflammatory demyelinating lesions, extensive mononuclear cell infiltration into the central nervous system (CNS), and loss of motor function. Despite improved understanding of the mechanisms by which MS is manifested, current treatment options for this disease – such as interferon (IFN)-1α, IFN-1β, glatiramer acetate, fingolimod, mitoxantrone, and natalizumab – have limited efficacy in providing symptomatic relief and complete remission, especially in patients affected with the chronic form of MS [31,32]. The application of adult stem cells as a potential treatment in MS patients is of interest, especially for patients who do not respond to the pharmacologic immunosuppression regimens with steroids or corticotropin.

Myelin oligodendrocyte glycoprotein (35–55)-induced experimental autoimmune encephalitis (EAE) in C57Bl/6J mice correlates with chronic, progressive MS and is an appropriate model to investigate the effects of treatment with BMSCs, ASCs, or SVF. While it has been reported that mesenchymal lineage stem cells from the bone marrow have a therapeutic impact in the EAE model, only one study to date has investigated murine ASCs, with no studies investigating the role of human ASCs or SVF [33]. The goal of this study was to directly compare human SVF cells with human BMSCs and ASCs as interventions for disease progression in the EAE model.

## Methods

### Cell isolation and culture

#### Bone-derived mesenchymal stem cells

MSCs from normal healthy donors were obtained from the Tulane Center for the Stem Cell Research and Regenerative Medicine (New Orleans, LA, USA). The cells were prepared from four individual donors with written, informed consent under protocols approved by Tulane Biomedical Institutional Review Board.

In brief, bone marrow aspirates were taken from the iliac crest of normal adult donors. Nucleated cells were isolated using a density gradient (Ficol-Paque; Amersham Pharmacia Biotech, Milwaukee, WI, USA) and resuspended in complete culture medium (CCM): α-modified Eagle’s medium (GIBCO/BRL, Grand Island, NY, USA); 20% FBS (lot selected for rapid growth; Atlanta Biologicals, Norcross, GA, USA); 100 units/ml penicillin (GIBCO/BRL); 100 μg/ml streptomycin (GIBCO/BRL); and 2 mM l-glutamine (GIBCO/BRL). Cells (4 × 10^7^ to 13 × 10^7^) were then plated in 20 ml medium in a 150-cm^2^ culture dish and incubated at 37°C with 5% humidified CO_2_. After 24 hours, nonadherent cells were removed. Adherent cells were washed twice with phosphate-buffered saline (PBS, pH 7.4) and incubated with fresh medium. After 5 to 7 days, the cells were harvested with 0.25% trypsin/1 mM ethylenediamine tetraacetic acid (EDTA) for about 5 minutes at 37°C and then replated at approximately 3 to 100 cells/cm^2^ in an inter-connecting flask system (Cell Factory; Nunc, Rochester, NY, USA). When the cultures reached 70% confluence, the cells (passage 1) were harvested with trypsin/EDTA, resuspended at 1 × 10^6^ cells/ml in α-modified Eagle’s medium with 5% dimethyl sulfoxide and 30% FBS, frozen in 1 ml aliquots overnight at -80°C, and then stored in liquid nitrogen. For cell expansion, frozen vials of passage-1 MSCs were thawed, plated in 25 ml complete MSC medium in a 150-cm^2^ culture dish (Nunc), and incubated at 37°C with 5% humidified CO_2_. After 24 hours, the medium was removed and adherent, viable cells were washed twice with PBS, harvested with EDTA, replated at 100 cells/cm^2^ in CCM, and incubated with a medium change every 3 to 4 days. For all experiments, subconfluent cells (≤70% confluent) between passages 2 and 6 were used.

#### Stromal vascular fraction

The SVF was obtained from the subcutaneous white adipose tissue from three donors undergoing elective liposuction procedures. Tissues were washed three to four times with PBS and suspended in an equal volume of PBS supplemented with 1% bovine serum and 0.1% collagenase type I (Worthington Biochemical Corporation, Lakewood, NJ, USA) prewarmed to 37°C. The tissue was placed in a shaking water bath at 37°C with continuous agitation for 60 minutes and centrifuged for 5 minutes at 300 to 500 × *g* at room temperature. The supernatant, containing mature adipocytes, was aspirated. The pellet was identified as the SVF cell population. Portions of the SVF cells were resuspended in cryopreservation medium (10% dimethyl sulfoxide, 10% Dulbecco’s modified Eagle’s medium/F-12 Ham’s, 80% FBS), frozen at -80°C in an ethanol-jacketed closed container, and subsequently stored in liquid nitrogen [34,35]. All protocols were reviewed and approved by the Pennington Biomedical Research Center Institutional Review Board and all human participants provided written informed consent.

Frozen vials of approximately 10^6^ SVF were thawed, suspended in 50 ml prewarmed CCM, centrifuged at 400 × *g* for 10 minutes, washed twice in Hank’s balanced salt solution (HBSS), and counted with a hemocytometer, as described previously [36].

#### Adipose-derived stem cells

Frozen vials of approximately 1 × 10^6^ ASCs were obtained from four separate donors from the Pennington Biomedical Research Center with written, informed consent under the same protocols approved by the Pennington Biomedical Research Center Institutional Review Board.

Vials were thawed, plated on 150 cm^2^ culture dishes (Nunc) in 25 ml CCM, and incubated at 37.5°C with 5% humidified CO_2_. After 24 hours, the media were removed and adherent, viable cells were washed twice with PBS, harvested with 0.25% trypsin/1 mM EDTA (Gibco), and replated at 100 cells/cm^2^ in CCM. Media was changed every 3 to 4 days. For all experiments, subconfluent cells (≤70% confluent) between passages 2 and 6 were used and grown under the same conditions as BMSCs.

### Experimental autoimmune encephalitis induction and treatment protocols

All animal experiments were approved by Tulane University School of Medicine’s Institutional Animal Care and Use Committee and were conducted in accordance with the US Public Health Service Policy on Human Care and Use of Laboratory Animals.

Female C57Bl/6J mice, 6 to 8 weeks old, were purchased from Charles River Laboratories (Wilmington, MA, USA). Chronic EAE was induced in these animals by subcutaneous immunization with 200 μl of 200 ng myelin oligodendrocyte glycoprotein (35–55) (Anaspec, San Diego, CA, USA) mixed 1:1 in complete Freund’s adjuvant with 8 mg/ml *Mycobacterium tuberculosis* H35RA (Difco, Detroit, MI, USA). About 100 μl was injected subcutaneously at each side of the base of the tail. Mice also received 100 μl of 200 ng pertussis toxin (List Biological Laboratories, Campbell, CA, USA) by intraperitoneal injection concomitantly and again 2 days later. Cells were pooled together per treatment (with approximately the same number of cells per donor) and 100 μl of 1 × 10^6^ cells suspended in HBSS were injected with a 27-gauge needle into the left side of the peritoneal cavity during EAE induction (day 0; Additional file 1). Sham-treated, EAE-induced mice received equal volumes of HBBS without cells.

### Clinical scoring and statistical analysis

Naïve mice (*n* = 12), EAE sham-treated mice (*n* = 12), EAE mice treated with BMSCs (*n* = 10), EAE mice treated with ASCs (*n* = 12), and EAE mice treated with SVF (*n* = 12) were monitored daily for clinical signs of disease by three independent, blinded investigators. Clinical scores were based on a scale of 0 to 5 (0, no disease; 1, limp tail (loss of tail tone); 2, hind limb weakness; 3, partial hind limb paralysis; 4, complete hind limb paralysis; and 5 moribund or dead). No animals were excluded from analysis. Clinical scores are presented as the mean ± standard error of the mean for each group, with dead animals given a score of 5 on the day of death and for the remainder of the experiment. Using GraphPad Prism version 4.0b (GraphPad Software Inc., San Diego, CA, USA, USA), statistical analysis on days 10 to 14 and again on days 26 to 30 was determined by one-way analysis of variance followed by pairwise comparisons of the mouse groups using Bonferroni *post-hoc* testing. Significance for the overall group effect and individual pairwise comparisons was defined as *P* < 0.05. The disease onset, disease incidence, and mean maximum scores were recorded for each mouse and expressed as the mean ± standard deviation. The cumulative disease score was calculated by summing the daily clinical score for each mouse during the course of observation.

### Tissue processing and histological analysis

Thirty days post disease induction, five animals per group were euthanized by exposure to CO_2_ and perfused with sterile PBS. Spinal cords were removed, postfixed in 10% formalin (Thermo Fisher Scientific, Waltham, MA, USA) and then embedded in paraffin. Sections were cut at 6 μm thickness on a microtome and stained for hematoxylin and eosin (Thermo Fisher) to reveal perivascular inflammatory infiltrates and luxol fast blue/cresyl violet (IHC World, Ellicott City, MD, USA) and toluidine blue (Thermo Fisher) for myelin detection. Histological analysis was performed on a Nikon Eclipse E800 (Nikon, Melville, NY, USA) microscope, acquired with Slidebook version 5.0 software (Olympus, Center Valley, PA, USA) and analyzed using Fiji/Image J software. Quantification for each stain was performed on nine random sections per animal and five animals per group. All images were analyzed by investigators that were blinded to the status of the animal. The demyelination score was measured by the ratio of area of intact myelin against the same values for the naïve group, which is set to 1. An index of cellular debris was determined by the percent of positive pixels divided by the percent of positive pixels for the naïve group, which is set to 1. The percent of inflammatory infiltrates was measured by the number of total cells (that is, cells 5 μm^2^) per field at 400× magnification.

### Cytokine detection

At 30 days post disease induction, blood was collected from all mice during intracardial perfusions. Cytokines were analyzed by enzyme-linked immunosorbent assay immunoassay (Life Technologies, Grand Island, NY, USA), according to the manufacturer’s instructions. Briefly, serum was added in 96-well precoated plates and incubated at room temperature. After washing, a specific polyclonal antibody followed by substrate solution was added, and the color development was measured at 450 nm on a fluorescent microplate reader (FLUOstar optima; BMG Labtech Inc., Durham, NC, USA). The concentration of cytokines was calculated using the standard curve. Statistical analysis using a two-tailed Student’s *t* test was performed to evaluated differences between groups. Significance was assumed if *P* ≤ 0.05.

## Results

### Intraperitoneal injection of human cells ameliorates myelin oligodendrocyte glycoprotein (35–55)-induced EAE

**Figure 1:**
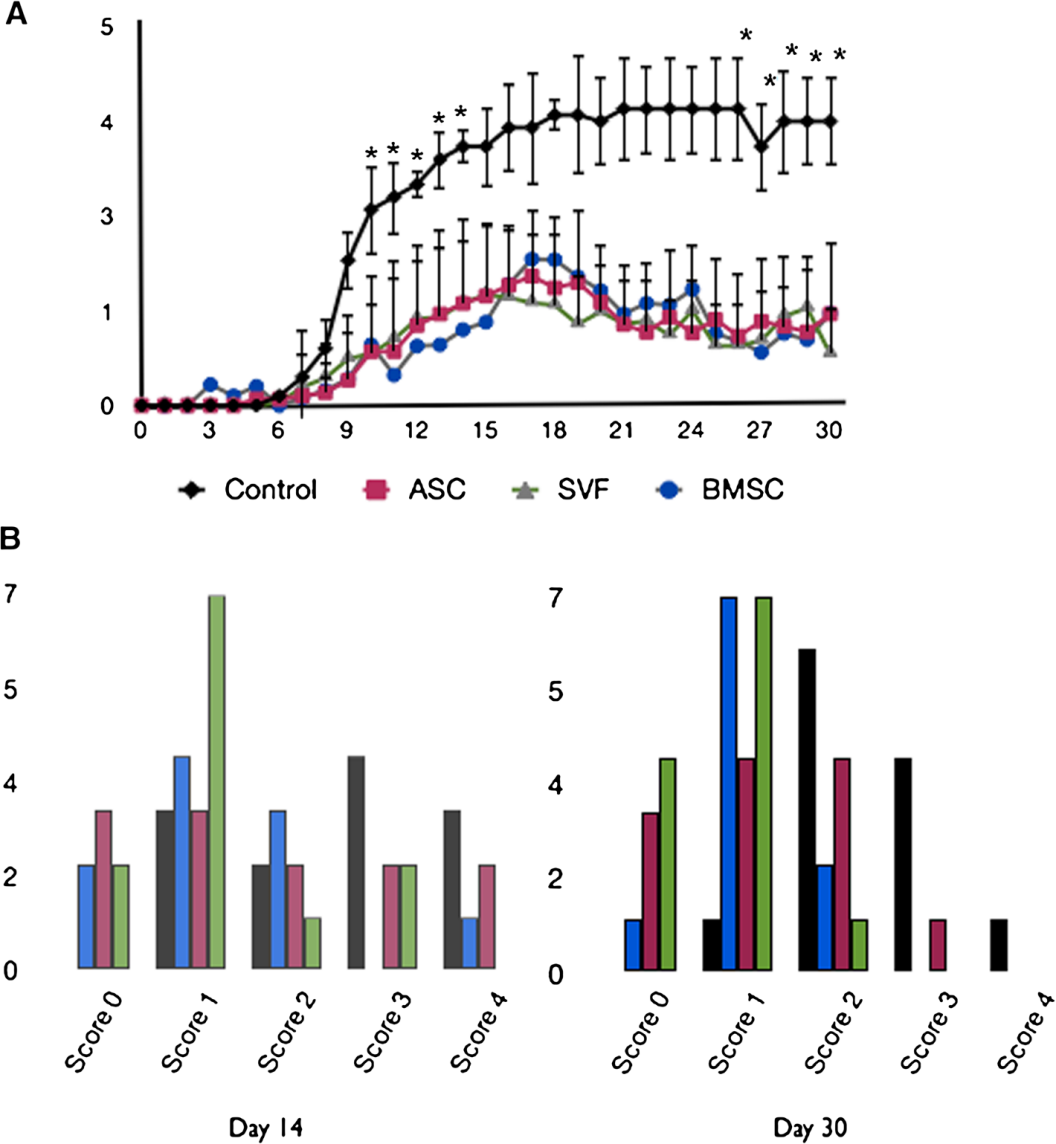
**Preventative administration of human cell therapy reduces the clinical score of myelin oligodendrocyte glycoprotein (35–55)-induced experimental autoimmune encephalitis.** Improved clinical scores were seen in bone-derived marrow stromal cell (BMSC)-treated (*n* = 10), adipose stem cell (ASC)-treated (*n* = 12), and stromal vascular fraction (SVF)-treated (*n* = 12) mice compared with controls (*n* = 12). **(A)** Values are means from three independent reviewers. Bars, ± standard error of the mean. *P ≤ 0.05, comparing controls and treated mice. **(B)** The clinical scores at day 14 days post disease induction show the distribution during peak disease, demonstrating that the majority of treated mice displayed less severe symptoms (left panel). The clinical scores at the end of the course demonstrate that the treated groups stably maintained their reduced state of disease (right panel).

To evaluate whether the human cells could affect disease progression in an autoimmune disease such as EAE, a preventative protocol was investigated in which cells were administered intraperitoneally at the time of disease induction. BMSC-treated, ASC-treated, and SVF-treated groups halted disease progression when compared with HBSS-treated controls, resulting in a statistically significant reduction of cumulative disease scores (Figure 1, Table 1). Although treatment groups developed the first clinical signs of disease at the same time as the control group, the mean maximum score was reduced in all treatment groups (Figure 1, Table 1). Furthermore, the disease incidence was significantly reduced in the ASC and SVF groups (75% and 83%, respectively) (Figure 1, Table 1). These data suggest that intraperitoneal injection of human BMSCs, ASCs, or SVF cells did not alter the timing of the onset of disease but did result in reduced disease severity. Moreover, it also indicates that the therapeutic effect is similar between all three treatment groups.

**Table 1:** Clinical–pathological features of experimental autoimmune encephalitis mice and treatment groups

**Treatment**	**Disease onset,DPI (range)**	**Disease incidence, *n*/*n *** **total (%)**	**Mean maximum score (range)**	**Cumulative disease score**	**Inflammatory area (%)**	**Demyelinated area (%)**	**Axonal loss (%)**
Control	9.0 ± 1.0 (7 to 10)	12/12 (100)	3.5 ± 0.5 (3 to 4)	30.4 (±9.57)	14.62 ± 6.6	0.68 ± 0.3^†^	6.1 ± 2.0^†^
BMSC	10.9 ± 1.9 (8 to 13)	10/10 (100)	2.3 ± 1.1 (1 to 4)	14.2 ± 11.8	7.3 ± 4.9	0.91 ± 0.3	1.5 ± 0.9
ASC	9.7 ± 0.9 (9 to 15)	9/12 (75)	2.6 ± 0.7 (1 to 4)	16.1 ± 7.2*	5.1 ± 4.8	0.75 ± 0.2	1.3 ± 1.5
SVF	9.3 ± 1.6 (2 to 3)	10/12 (83)	2.6 ± 0.7 (1 to 4)	10.8 ± 6.4*	6.7 ± 4.4	0.76 ± 0.2	3.1 ± 0.9

ASC, adipose stem cell; BMSC, bone-derived marrow stromal cell; DPI, days post disease induction; SVF, stromal vascular fraction. *P ≤ 0.05 between control group and ASC-treated group and SVF-treated group. ^†^*P* ≤ 0.05 between control group and experimental groups.

### Pathological features are diminished with cell therapy

**Figure 2:**
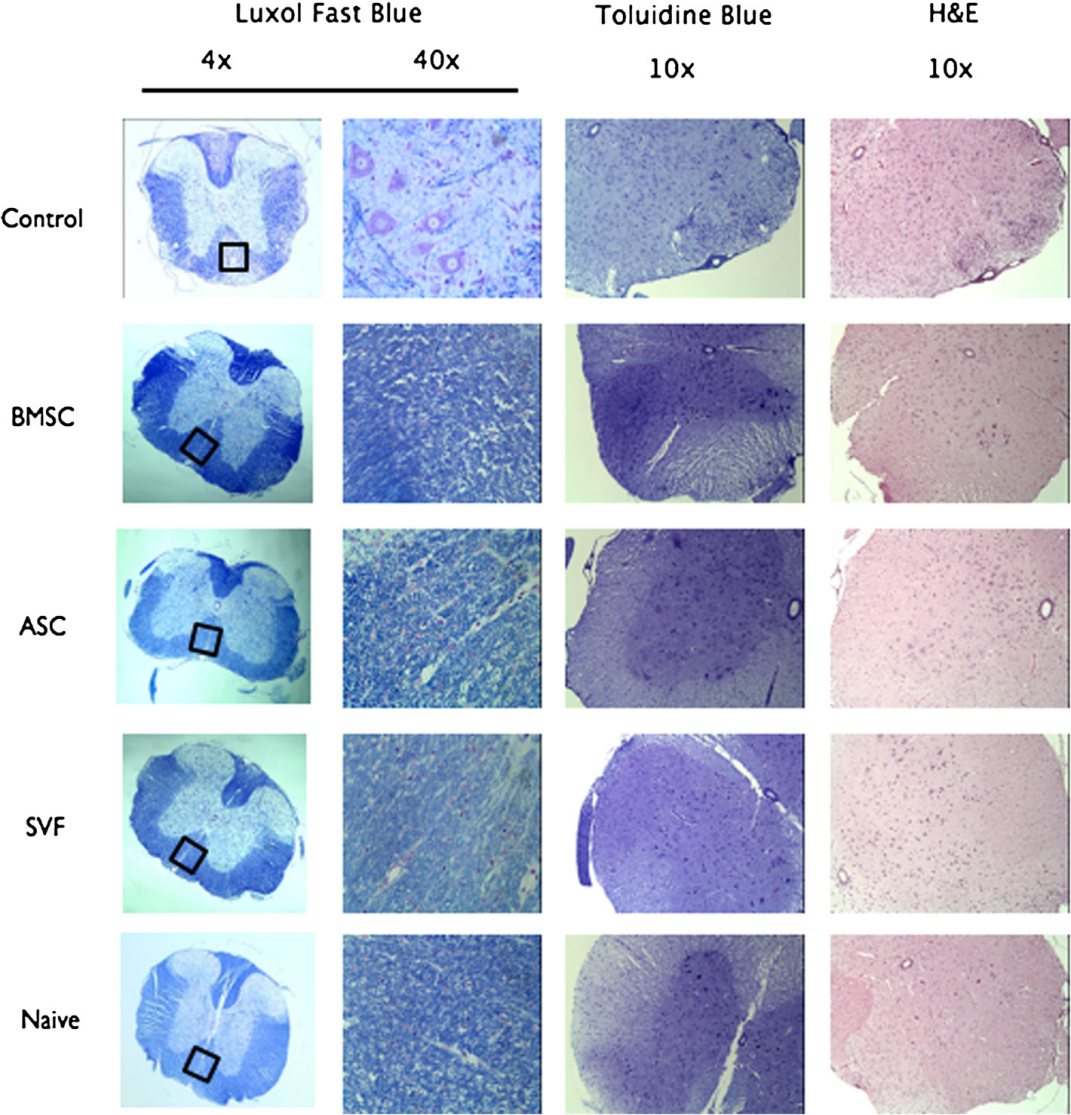
**Treatment with human cell therapy reduces cellular infiltration and tissue damage in experimental autoimmune encephalitis.** Spinal cords from Hank’s balanced salt solution (HBSS)-treated mice, bone-derived marrow stromal cell (BMSC)-treated mice, adipose stem cell (ASC)-treated mice, stromal vascular fraction (SVF)-treated mice, and naïve mice were obtained after euthanasia at 30 days post disease induction and processed for histological staining using luxol fast blue (LFB), toluidine blue (TB), and hematoxylin and eosin (H&E). LFB staining identified multiple areas of demyelination in HBSS-treated experimental autoimmune encephalitis **(**EAE) mice but only scattered foci in treatment groups. Similarly, sections labeled with TB showed increased myelin debris and greater numbers of demyelinated axons in the control mice compared with naïve or treated mice. Comparisons of the H&E images show a decrease in the number of infiltrating immune cells in the spinal cord after administration of both ASC and SVF.

To determine whether the reduced disease severity correlated with pathological features, spinal cords from naïve mice, HBSS-treated mice, BMSC-treated mice, ASC-treated mice, and SVF-treated EAE mice were stained with luxol fast blue to analyze regions of demyelination. Quantification was performed on nine sections per animal and five animals per group. All images were analyzed by investigators blinded to the status of the animal. Indexes were normalized to the average value obtained in naïve mice (set to 1). The extent of demyelinated regions was reduced in all treatment groups compared with HBSS-treated mice (Figure 2, Table 1). Similarly, all treatment groups were stained with toluidine blue and demonstrated reduced myelin breakdown products and debris compared with controls (an index of 1.5 ± 0.9 in BMSC-treated mice, 1.3 ± 1.5 in ASC-treated mice, and 3.1 ± 0.9 in SVF-treated mice compared with 6.1 ± 2.0 in HBSS-treated mice), consistent with the neuroprotective effect of all therapies in EAE (Figure 2, Table 1). The influx of immune infiltrates was measured by hematoxylin and eosin staining of spinal cords. The percentage of infiltrating cells was significantly decreased in animals treated with BMSCs (7.3 ± 4.9%), ASCs (5.1 ± 4.8%) or SVF (6.7 ± 4.4%) compared with HBSS-treated mice (14.6 ± 6.6%), indicating that all cell treatments effectively reduced the level of inflammatory infiltrates into the CNS during the course of EAE disease (Figure 2, Table 1).

### The stromal vascular fraction and adipose stem cells both suppressed IFNγ and interleukin-12 in the sera of EAE mice

**Figure 3:**
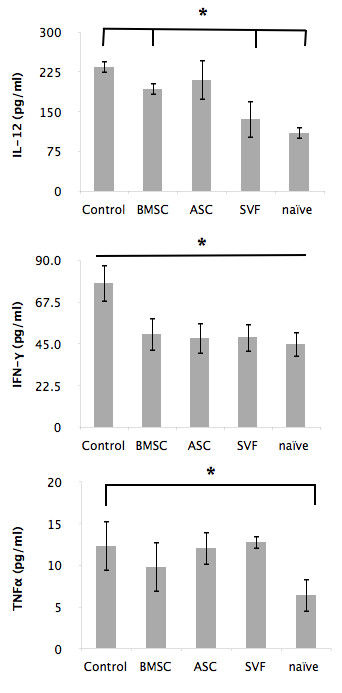
**Serum levels of inflammatory cytokines in naïve and treated mice with experimental autoimmune encephalitis.** At 30 days post disease induction, blood was collected from mice during intracardial perfusion and analyzed by enzyme-linked immunosorbent assay immunoassay for interleukin (IL)-12, interferon (IFN)γ, and tumor necrosis factor alpha (TNFα). IL-12 levels were decreased in bone-derived marrow stromal cell (BMSC)-treated mice and further decreased in stromal vascular fraction (SVF)-treated mice compared with control mice (middle panel), while adipose stem cell (ASC)-treated mice were not significantly decreased. All treatment groups had a similar decrease in levels of IFNγ (upper panel). No difference was seen in TNFα (bottom panel). Bars, ± standard deviation, *P ≤ 0.05.

Tumor necrosis factor alpha (TNFα), interleukin (IL)-12, and IFNγ are cytokines shown to be responsible for T-helper type-1 (Th1) cell stimulation that play a central role in the pathogenesis of MS and EAE [33,37]. To determine whether the protective effects of the cell therapy were related to these reduced levels of these factors, cytokine levels in the sera were assayed by enzyme-linked immunosorbent assay. Although the IFNγ sera levels of BMSC-treated mice (50.09 ± 9.0 pg/ml), ASC-treated mice (48.12 ± 8.5 pg/ml) and SVF-treated mice (48.59 ± 7.8 pg/ml) were lower than the untreated control (77.71 ± 10.0 pg/ml) (*P* < 0.01), the cell-treated groups were not statistically significant among each other (Figure 3). The IL-12 sera levels were significantly decreased in the BMSC-treated mice (193.45 ± 11.6 pg/ml) and further decreased in the SVF-treated mice (136.23 ± 35.1 pg/ml) compared with untreated control mice (235.18 ± 12.0 pg/ml) (*P* < 0.01), but the ASC-treated mice (210.42 ± 37.8 pg/ml) were unchanged (Figure 3). The levels of TNFα were not significantly reduced in any experimental group at this time point (Figure 3). Collectively, these data suggest that all three treatment groups have a similar ability to reduce levels of IFNγ, an important Th1 inflammatory cytokine, in the sera, while BMSC and SVF treatment can also decrease IL-12 levels.

## Discussion

MSCs are a promising therapy for the treatment of CNS-related autoimmune diseases due to their immunomodulatory and neuroprotective effects. However, the source and availability of MSCs is becoming a crucial issue for their clinical application. BMSCs, the most studied MSCs, have demonstrated the ability to ameliorate both chronic and relapsing-remitting EAE [38-40]. Although BMSCs have demonstrated promising results, the invasive nature of bone marrow biopsies may limit their practicality for wider clinical applications. Adipose tissue has become an appealing cell source for regenerative medicine and tissue engineering, since it contains a large number of ASCs, is easy to obtain in large volumes, and is easily accessible [41-44]. ASCs have been shown to hold many of the same properties as BMSCs, such as the ability to differentiate, inhibit T-cell activation and proliferation, produce anti-inflammatory molecules, and aid in tissue repair through the secretion of cytokines [45].

Despite the promising potential of ASCs, the need for *ex vivo* cellular expansion still presents a significant challenge for human applications. The uncultured counterpart of ASCs, the SVF, is a particularly promising candidate for regenerative medicine because the cells can be isolated within hours of obtaining the lipoaspirate and no culture expansion of the cells is required, which would reduce any potential risks associated with growing cells *in vitro* and remove the need for specialized laboratories. In addition to the clinical risks of *ex vivo* expansion, the variables used in cell culture, such as percent and source of serum used, type of basal media, media supplements, culture surface substrate, cell seeding density, passage number, and confluency of culture, are undoubtedly giving way to contrasting and confusing results in research.

Adipose tissue comprises one of the largest organs in the body and serves as an important endocrine organ regulating many facets of homeostasis [26,46,47]. Comprised of mature adipocytes and other nonadipocyte cells, adipose tissue can be manually disrupted and/or treated with collagenase to isolate the SVF. Although not a fully defined cell population, the SVF includes vascular smooth muscle cells, fibroblasts, mast cells, macrophages, lymphocytes, endothelial cells, pre-adipocytes, and ASCs [48-52].

SVF cells have been used clinically to treat acute and chronic diseases afflicting a range of tissues and organs, including soft tissue defects, breast reconstruction, and autoimmune diseases such as graft-versus-host-disease, rheumatoid arthritis, and Crohn’s disease [27,30,41,46,53]. To date only one group has demonstrated improved function in MS patients treated with SVF; however, mechanisms behind the improvements were not explored [30]. Similarly, only one study showed that culture-expanded murine ASCs had a significant beneficial effect on chronic EAE by acting simultaneously in the lymphoid organs as well as the inflamed CNS and causing a dramatic change in antigen-specific T cells [33]. This present study is the first to investigate human ASCs and SVF cells in the treatment of EAE. Although SVF cells had similar mean maximum disease scores and time of disease onset to ASCs, the SVF had lower cumulative disease score. We previously compared the ability of murine SVF with ASCs in the same EAE model and showed that the SVF effectively inhibited disease severity and was statistically more effective than ASCs [54]. Unlike the human SVF, which had a disease incidence of 10 out of 12 mice (Table 1), the murine SVF only had 3 out of 12 mice demonstrate clinical signs [54]. EAE mice treated with human SVF had a disease onset of 9.3 ± 1.6 (Table 1) while EAE mice treated with murine SVF had a disease onset of 15 ± 4.5 days post disease induction (unpublished findings). The disparate results between human and mouse SVF may be due to species differences or some undefined mechanism(s). The current results show that uncultured SVF can ameliorate clinical symptoms as well as reduce spinal cord inflammation, demyelination, and axonal damage without the dangers associated with *ex vivo* cellular expansion.

Furthermore, SVF treatment had a similar effect on the systemic immune response in EAE mice. IFNγ is a cytokine associated with a number of autoinflammatory and autoimmune diseases, due to its role in Th1 cell stimulation, differentiation, and function via STAT1 and STAT4 pathways [37]. These autoreactive T cells play a central role in the direct regulation of T-cell activation and survival during autoimmune inflammation in the pathogenesis of MS and EAE [37,55]. In this study, IFNγ was reduced comparably between treatment groups. These results point to the ability of SVF cells to play an effector role similar to that of both ASCs and BMSCs, which would occur during the early inflammatory phase of disease, supporting the possibility that uncultured SVF cells could also affect the generation of encephalitogenic effector T cells. Although BMSCs have been shown to reduce levels of IFNγ by direct contact, it is unclear whether ASCs and SVF cells utilize the same mechanism [56]. Although the mechanisms mediating such effects are still only partially understood, it is likely that they involve both direct cell-to-cell contact and paracrine signaling through soluble factors.

In addition to IFNγ, IL-12 is responsible for Th1 cell stimulation, differentiation, and function and plays a central role in the pathology of MS [37]. In this study, IL-12 was reduced in the BMSC-treated group and further reduced in the SVF-treated group. Although this is the first study to show that SVF treatment reduced the levels of IL-12, BMSCs have been shown to reduce levels of IL-12 in a chronic EAE model [56,57]. Interestingly, murine BMSCs were shown to exert opposing effects on Th1 cells depending on the time of disease onset and the level of effector T-cell activation, suppressing all T cells when administered early during T-cell activation and able to decrease IFNγ and increase IL-17 once T cells become activated [58]. These results indicate that IL-12 may play an important mechanistic role during the increased potency of SVF-based therapy. It is possible that the SVF cells, beyond their *ex vivo* expanded ASC counterpart, have the ability to further reduce the level of effector T-cell activation, keeping the disease in a more naïve state by reducing IL-12, and therefore Th1 stimulation and differentiation. Whether this is a result of the ASCs being uncultured and retaining more of their *in vivo* properties, is a result of the SVF being administered in a heterogeneous population, or is a product resulting from the interaction of ASCs with one of the other cell types present remains unclear.

Although BMSCs have been shown to reduce TNFα levels by direct contact *in vitro*, this study showed that intraperitoneal injection of neither BMSCs, ASCs, or SVF cells affected TNFα levels in an EAE model [48]. This may be due to the BMSCs being injected locally, murine BMSCs utilizing a different mechanism, or the studies being administered at a different time points during T-cell activation and differentiation. This also reiterates the speculation that the timing of stem cell interaction with T cells may drastically change immunomodulatory results and, therefore, disease progression.

These results point to the ability of all three cell types to play an effector role, which would occur during the early inflammatory phase of disease. Although the mechanisms mediating such effects are still only partially understood, it is likely that they involve both direct cell-to-cell contact and paracrine signaling through soluble factors. Further work needs to address whether the treatments utilized similar or unique mechanisms. This work also reiterates the speculation that the timing of cell therapy may drastically change immunomodulatory results and, therefore, disease progression. Further work needs to address cell therapy efficacy and cytokine response during the course of the disease, paying particular attention to cytokines that change quickly after cell treatment, such as T-helper type-17 cells. Although these data demonstrate that BMSCs, ASCs, and the SVF could affect the generation of encephalitogenic effector T cells, whether they can affect viability and function of the encephalitogenic effector T cells in established disease still needs to be determined.

## Conclusion

There is an increasing interest in the biology and therapeutic potential of the SVF due to the direct and rapid isolation procedure in a xenobiotic-reduced environment [41,42,51]. Whether the potency of the SVF is a result of the fact that the cells do not undergo extensive *ex vivo* culture because the injected cells are a mix of ASCs in combination with lymphohematopoietic and other cell types or a combination of both needs to be further determined. Regardless, the findings demonstrate that when compared with *ex vivo* expanded ASCs, SVF cells are equivalent to cultured cells in their ability to ameliorate EAE disease progression, and further reduce Th1-type cytokines. Moreover, the SVF is simple and straightforward to isolate. The SVF cells have relevant therapeutic potential in an animal model of chronic MS and might represent a valuable tool for stem cell-based therapy in chronic inflammatory disease of the CNS.

## Abbreviations

ASC: adipose stem cell; BMSC: bone-derived marrow stromal cell; CCM: complete culture medium; CNS: central nervous system; EAE: experimental autoimmune encephalitis; EDTA: ethylenediamine tetraacetic acid; FBS: fetal bovine serum; HBSS: Hank’s balanced salt solution; IFN: interferon; IL: interleukin; MS: multiple sclerosis; MSC: mesenchymal stromal/stem cell; PBS: phosphate-buffered solution; SVF: stromal vascular fraction; Th1: T-helper type 1; TNF: tumor necrosis factor.

## Competing interests

JMG is the co-founder, co-owner and Chief Scientific Officer and FSS is an employee at LaCell LLC, a private, for-profit biotechnology company focusing on adult stem cell products from adipose and other tissues. The remaining authors declare that they have no competing interests.

## Authors’ contributions

JAS participated in the design and coordination of the study, and drafted the manuscript. XZ, ACP, and SZ evaluated animals. CM carried out the immunoassays and participated in microscopy. SAS evaluated animals and participated in microscopy. MMB participated in microscopy and cell culture. FSS and BAS participated in cell culture. ALS participated in harvesting of organs and histology. JMG participated in the design of the study. BAB conceived of the study, participated in its design and coordination, and helped draft the manuscript. TAS participated in harvesting of organs and histology. All authors read and approved the final manuscript.

## Supplementary Material

Additional file 1: Figure S1 Showing a schematic of the EAE induction and treatment protocol. Chronic EAE was induced in mice by subcutaneous immunization with 200 μl of 200 ng myelin oligodendrocyte glycoprotein (35–55) mixed 1:1 in complete Freund’s adjuvant with 8 mg/ml *Mycobacterium tuberculosis* H35RA. About 100 μl was injected subcutaneously at each side of the base of the tail on day 0. Mice also received 100 μl of 200 ng pertussis toxin on day 0 and a booster on day 2. Cells were administered in a preventative disease setting by injecting 100 μl of 1 × 10^6^ cells suspended in HBSS into the left side of the peritoneal cavity during EAE induction (day 0). Click here for file
