# Supplementary figures and images for: Comparison of human adult stem cells from adipose tissue and bone marrow in the treatment of experimental autoimmune encephalomyelitis
Source: Stem Cell Res Ther. 2014 Jan 9;5(1):2. doi: 10.1186/scrt391 (PMC4054950; doi:10.1186/scrt391)

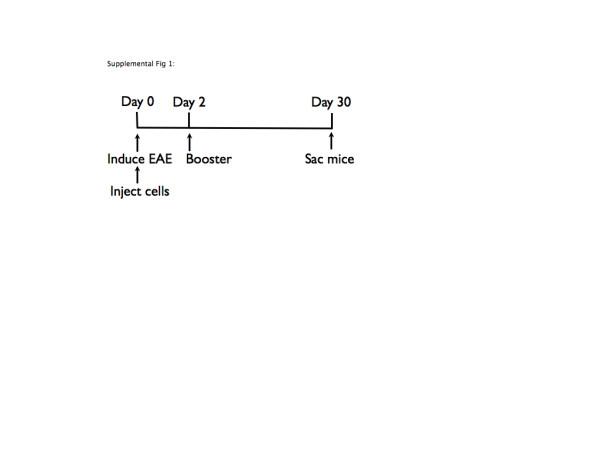

Supplement: Additional file 1: Figure S1 — Showing a schematic of the EAE induction and treatment protocol. Chronic EAE was induced in mice by subcutaneous immunization with 200 μl of 200 ng myelin oligodendrocyte glycoprotein (35–55) mixed 1:1 in complete Freund’s adjuvant with 8 mg/ml Mycobacterium tuberculosis H35RA. About 100 μl was injected subcutaneously at each side of the base of the tail on day 0. Mice also received 100 μl of 200 ng pertussis toxin on day 0 and a booster on day 2. Cells were administered in a preventative disease setting by injecting 100 μl of 1 × 10^6^ cells suspended in HBSS into the left side of the peritoneal cavity during EAE induction (day 0). [file scrt391-S1.jpeg]
